# Transdiagnostic Comparison of Anticipated Hedonic Deficits: A Three‐Level Meta‐Analysis Across Schizophrenia, Depression, and Their Subclinical Individuals

**DOI:** 10.1002/pchj.70094

**Published:** 2026-04-02

**Authors:** Yan Gao, Jin‐ting Yu, Simon S. Y. Lui, Tian‐xiao Yang, Raymond C. K. Chan

**Affiliations:** ^1^ Neuropsychology and Applied Cognitive Neuroscience Laboratory, State Key Laboratory of Cognitive Science and Mental Health Institute of Psychology, Chinese Academy of Sciences Beijing China; ^2^ Department of Psychology The University of Chinese Academy of Sciences Beijing China; ^3^ School of Psychology, Beijing Sport University Beijing China; ^4^ Department of Psychiatry, School of Clinical Medicine The University of Hong Kong Hong Kong Special Administrative Region China

**Keywords:** anhedonia, anticipated displeasure, anticipated pleasure, depression, schizophrenia

## Abstract

Anhedonia, a transdiagnostic symptom for schizophrenia and depression, exists in subclinical individuals at risk of the two disorders. Prior meta‐analytic reviews seldom considered both anticipated pleasure and anticipated displeasure. We conducted a three‐level meta‐analysis on anticipated pleasure and displeasure in people with schizophrenia and depression, and their subclinical counterparts. Clinical and subclinical individuals of the schizophrenia spectrum reported less anticipated pleasure than controls (*k* = 37, 1464 participants, *g* = −0.22, *p* = 0.032), but reported similar anticipated displeasure as controls (*k* = 20, 769 participants, *g* = 0.09, *p* = 0.345). Clinical and subclinical individuals of depression anticipated less pleasure (*k* = 21, 1162 participants, *g* = −0.62, *p* = 0.003) and more displeasure (*k* = 15, 954 participants, *g* = 0.82, *p* = 0.033) than controls. Comparisons of the schizophrenia and depression samples yielded no significant difference for effect sizes of either anticipated pleasure or anticipated displeasure. For schizophrenia spectrum, heterogeneity of anticipated pleasure was explained by sociality of anticipated stimuli. For participants with depression, higher severity of depressive symptoms were associated with larger between‐group effects on anticipated pleasure and displeasure. After accounting for publication bias, the between‐group effects remained of a similar magnitude. We elucidated the patterns of impaired anticipated emotions in clinical and subclinical samples of the schizophrenia and depression. Social anticipated pleasure may be a potential screening target for schizophrenia, while impaired anticipated emotions may serve as a marker for depression.

## 1 Introduction

Anhedonia refers to the diminished capacity to experience pleasure, and is the hallmark symptom of both depression and schizophrenia. In depression, anhedonia is characterized by a loss of interest and pleasure in activities that were once enjoyable. In schizophrenia, anhedonia constitutes negative symptoms (APA 2013). Anhedonia in depression is marked by deficits in anticipatory pleasure as well as the integration of reward‐associated information, while anhedonia in schizophrenia is linked to neurocognitive impairments in the representation of reward value (Lambert et al. 2018; Liang et al. 2022). Anhedonia is a risk factor for the onset of depression (Morgan et al. 2013), schizophrenia (Gooding et al. 2005; Kwapil 1998), and other psychotic disorders (Radua et al. 2018).

Research on anticipatory anhedonia has attracted much interest (Gard et al. 2006, 2007). Anticipatory pleasure includes the emotion one expects to experience from future events (anticipated emotion) and the positive emotions felt while imagining a future event (anticipatory affect) (Frost and Strauss 2016; Zhang et al. 2020). A meta‐analysis study revealed that both patients with schizophrenia and depression reported less anticipatory pleasure than healthy people, as assessed by self‐report scales and laboratory tasks (Hallford and Sharma 2019). In the general population, individuals who display symptoms similar to clinical patients but do not meet the full diagnostic criteria are regarded as subclinical samples (Phillips and Seidman 2008). However, this meta‐analysis study did not include subclinical samples and combined the studies of the anticipatory and anticipated pleasure, without specifically focusing on anticipated pleasure.

Indeed, previous empirical or meta‐analytic studies largely focused on anticipatory pleasure, but neglected the construct of anticipated emotions (Hallford and Sharma 2019; Moore et al. 2019; Visser et al. 2020; Zhang et al. 2020). Anticipated emotions include both anticipated pleasure and displeasure (Zhang et al. 2020), and more intense anticipated displeasure was associated with psychopathology (Rizeq 2024). Examining individuals' anticipated emotions of both positive and negative future events can provide a more comprehensive research framework for understanding symptoms and mechanisms of psychiatric disorders. A recent review synthesized the methodological approaches used in measuring anticipated emotions within the context of psychopathology, as well as the associations between psychotic symptoms and anticipated emotions (Rizeq 2024). Previous studies on anticipated pleasure and displeasure in clinical and subclinical individuals of the schizophrenia spectrum have yielded mixed results (Moore et al. 2019; Pillny et al. 2020; Zhang et al. 2023). However, the majority of studies found people with clinical and subclinical depression exhibited reduced anticipated pleasure (Mathersul and Ruscio 2020; Sun et al. 2022; Zetsche et al. 2019) and elevated anticipated displeasure (Hallford 2019; Thompson et al. 2017).

Importantly, previous meta‐analysis studies have not integrated the transdiagnostic and subclinical perspectives of psychiatric disorders (Hallford and Sharma 2019; Visser et al. 2020). The transdiagnostic approach facilitates comparative research among psychiatric disorders to better clarify the shared and distinct mechanisms and identify potential common or unique targets of treatments (Dalgleish et al. 2020; Nolen‐Hoeksema and Watkins 2011). Studying subclinical populations provides an opportunity to identify early vulnerability markers and understand the developmental trajectory of mental disorders before the onset of full‐blown illness, thereby informing targeted prevention strategies (Buntrock et al. 2024).

Our study therefore attempted to summarize the current divergent results using meta‐analysis, and to examine the anticipated pleasure and displeasure using the transdiagnostic (schizophrenia and depression) and the spectrum (clinical and subclinical) approaches. We tested whether anticipated pleasure and displeasure in samples within schizophrenia and depression groups would differ from that in healthy people. Similar to previous meta‐analytic studies (Hallford and Sharma 2019; Visser et al. 2020), we also examined the moderator effects of study design and sample characteristics on anticipated pleasure and displeasure.

## 2 Methods

### 2.1 Search Strategy

The review was conducted in accordance with the PRISMA guidelines (Liberati et al. 2009), and preregistered with PROSPERO (Registration number: CRD42023452684). We conducted a comprehensive search of electronic databases on March 3rd, 2025, including PubMed, PsycINFO, Web of Science, Elsevier, and ProQuest Dissertations and Theses. The keywords for searching were (“affective forecast*” OR “emotional forecast*” OR “anticipatory emotion*” OR “anticipated emotion*” OR “anticipatory pleasure” OR “anticipatory displeasure” OR “anticipated pleasure” OR “anticipated displeasure”) AND (schizo* OR depressi*). We limited the search to peer‐reviewed articles or dissertations that were published in English and involved human subjects. Manual search on the reference lists of all the identified articles was used to include eligible studies.

### 2.2 Study Selection

Inclusion criteria were: (1) comparative studies of clinical/subclinical group versus controls; (2) studies including people with schizophrenia or their subclinical individuals (individuals with high schizotypy traits), and people with depression or their subclinical individuals (individuals with high depression scores); (3) studies having measured anticipated pleasure and displeasure in relation to future events (but not anticipatory pleasure); (4) sufficient information regarding the methodology and the results for extraction, or which can be obtained from the authors. Exclusion criteria were: (1) review articles, meta‐analyses, editorials, or conference abstracts, (2) participants having psychiatric conditions other than schizophrenia or depression (e.g., bipolar disorder, autism, anxiety, etc.). Two authors (Y.G., J.‐T.Y.) independently screened the retrieved articles based on the inclusion and exclusion criteria, and discrepancies were resolved through discussion.

### 2.3 Data Extraction

We extracted the following information from the included articles: study characteristics (author, title, publication year, study region), sample characteristics (including sample size, age, percentage of females (%Female), percentage of non‐Caucasian participants (%Non‐Caucasian), education year), and clinical characteristics (including duration of illness, proportion of people with schizophrenia prescribed typical antipsychotics, chlorpromazine equivalent doses, negative/depressive symptom severity, percentage of antidepressants prescribed to people with depression). Detailed calculation methods for symptom severity are presented in Appendix S1: Section 1. Furthermore, we classified the following categorical variables as additional study characteristics: clinical status (participants involved clinical or subclinical samples), assessment method for anticipated emotions (i.e., type of the task: questionnaire format, laboratory‐based task, or experience sampling task), sociality of anticipated stimuli (social stimuli: future stimuli involving other people; non‐social stimuli: future stimuli involving participants self or unrelated to people, such as future monetary rewards), hypotheticality of anticipated stimuli (hypothetical or actual future events), and temporal distance of anticipated stimuli (immediate: next few hours; distant: next day or later).

We applied the Newcastle‐Ottawa Scale (NOS), which employs a star system to evaluate various criteria and determine the data quality of studies (Wells et al. 2014). Following previous studies (Hallford and Sharma 2019; Stang 2010), we rated the quality of identified studies on a scale ranging from 0 to 7 on the NOS. Higher scores indicate better quality of the study.

Data were extracted independently and confirmed by two authors (Y.G., J.‐T.Y.). Any discrepancies were discussed by both authors until a consensus was reached. Then, we calculated the effect size for anticipated emotions, using the means, standard deviations, *t* statistics, and *p* values reported in the included articles.

### 2.4 Statistical Analysis

We calculated Hedges' *g* as the metric for effect size. We applied three‐level random‐effects models to synthesize the effect sizes across studies and to conduct moderator analyses. The three different sources of variance were modeled: (1) sampling variance of the effect sizes (Level 1), (2) variance between effect sizes extracted from the same study (Level 2), and (3) variance between studies (Level 3). The analyses were conducted separately for the schizophrenia and the depression groups. Detailed information is provided in Appendix S1: Section 2.

To address possible publication bias, we applied the following three methods. First, we used the funnel plots (Torgerson 2006). Second, we conducted Egger's test to detect funnel plot asymmetry (Egger et al. 1997). Third, when Egger's test indicated significance, we applied the trim‐and‐fill method to assess the overestimation or underestimation of the true overall effect size (Duval and Tweedie 2000).

The metafor package in the R environment (version 4.2.2) was used for analysis. The model parameters were estimated using the restricted maximum likelihood method. R syntax was scribed according to Assink and Wibbelink (2016). A two‐tailed *p* value smaller than 0.05 was considered to be statistically significant.

## 3 Results

### 3.1 Study Characteristics

**FIGURE 1:**
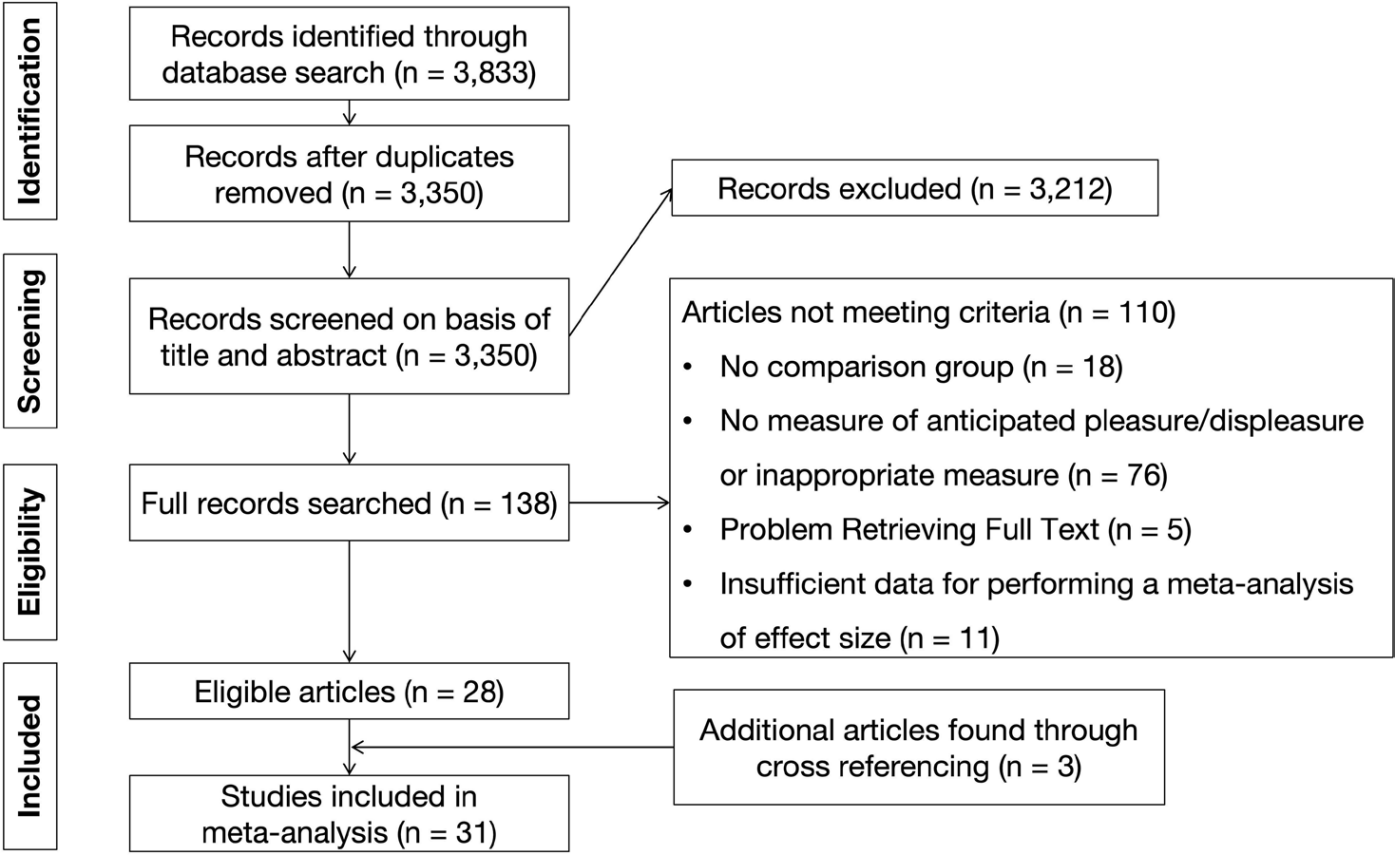
Flow diagram of study selection process.

Figure 1 presents the flow chart for study selection process, which identified 31 articles. As shown in Table 1, 19 of the included studies involved people with schizophrenia or subclinical individuals of the schizophrenia spectrum, and 10 involved people with depression or subclinical individuals with depression, and 2 studies involved subclinical samples of both the schizophrenia and depression (Pu et al. 2024; Zhang et al. 2021). Most of the studies were conducted in the United States (*n* = 16; 51.61%) or China (*n* = 10; 32.26%). European studies appeared to be under‐represented: three studies from Germany (*n* = 4; 12.90%) and one study from Greece (*n* = 1; 3.22%). The NOS scores ranged from 3 to 6 with a mean score of 3.73 (SD = 0.81).

**TABLE 1:** Characteristics of included studies.

Study (year)	Clinical status	Inclusion measure	Study group (*n*)	Control group (*n*)	Country	Task type	Sociality of stimuli	Mean age	% Female	%Non‐Caucasian
Schizophrenia and subclinical participants										
Campellone and Kring (2018)	Clinical	DSM‐IV	32	29	USA	Lab‐based	Social	46.88	49.18	46.88
Campellone et al. (2018)	Clinical	DSM‐IV	64	26	USA	Lab‐based	Social	23.64	30.00	52.24
Demmin et al. (2020)	Clinical	DSM‐5	15	15	USA	Lab‐based	Non‐social	38.17	20.00	53.33
Engel et al. (2016)	Clinical	DSM‐IV	40	40	Germany	Lab‐based		31.39	48.75	
Gard et al. (2007)	Clinical	DSM‐IV	15	12	USA	ESM		39.85	44.44	55.56
Gard et al. (2014)	Clinical	DSM‐IV	47	41	USA	ESM		38.28	30.68	55.68
Martin et al. (2019)	Clinical	DSM‐5	16	30	USA	Lab‐based	Social	44.74	54.35	54.35
Moore et al. (2019)	Subclinical	RSAS	21	23	USA	Lab‐based	Social	20.14	90.90	84.09
Moran et al. (2019)	Clinical	DSM‐IV	28	30	USA	Lab‐based	Non‐social	36.30	29.90	65.52
Pillny et al. (2020)	Clinical	DSM‐5	35	36	Germany	ESM	Both	34.65	63.75	
Pillny et al. (2024)	Clinical	DSM‐5	43	43	Germany	ESM		41.00	36.04	
Pu et al. (2024)	Subclinical	CSAS	35	47	China	Lab‐based	Both	20.59	67.07	100.00
Trémeau et al. (2010)	Clinical	DSM‐IV	70	35	USA	Lab‐based	Non‐social	37.13	17.13	6 4.56

Study (year)	Clinical status	Inclusion measure	Study group (*n*)	Control group (*n*)	Country	Task type	Sociality of stimuli	Mean age	% Female	%Non‐Caucasian
Schizophrenia and subclinical participants										
Trémeau et al. (2014)	Clinical	DSM‐IV	49	25	USA	Lab‐based	Non‐social	39.49	21.35	
Xie et al. (2014)	Subclinical	CSAS	28	38	China	Lab‐based	Both	20.71	63.63	100.00
Yan et al. (2019)	Clinical	DSM‐IV	44	46	China	Lab‐based	Non‐social	26.89	53.33	100.00
	Subclinical	SPQ	30	35	China	Lab‐based	Non‐social	20.56	55.39	100.00
Yang et al. (2021)	Subclinical	CSAS	49	33	China	Lab‐based		22.15	64.64	100.00
Zhang et al. (2020)	Subclinical	CSAS	40	46	China	Lab‐based	Both	21.33	69.77	100.00
Zhang et al. (2021)	Subclinical	SPQ	25	34	China	Lab‐based		22.98	59.32	100.00
Zhang et al. (2023)	Clinical	DSM‐5	47	47	China	Questionnaire	Both	32.08	55.32	100.00
Zhang et al. (2025)	Clinical	CSAS	28	32	China	ESM	Both	19.83	70.00	100.00

Study (Year)	Clinical status	Inclusion measure	Study group (*n*)	Control group (*n*)	Country	Task type	Sociality of stimuli	Mean age	% Female	%Non‐Caucasian
Depression and subclinical participants										
Li et al. (2019)	Subclinical	BDI	49	51	China	ESM		20.51	84.00	100.00
MacLeod and Salaminiou (2001)	Clinical	DSM‐IV	22	22	Greece	Lab‐based		57.00	45.45	
Marroquín et al. (2013)	Subclinical	BDI	154	135	USA	Lab‐based		20.20	71.97	57.40
Marroquín (2015)	Subclinical	BDI	77	84	USA	Lab‐based		20.90	70.00	48.00
Mathersul and Ruscio (2020)	Clinical	DSM‐IV	38	33	USA	Lab‐based		32.77	69.05	45.71
Pu et al. (2024)	Subclinical	BDI, PHQ‐9	53	47	China	Lab‐based	Both	20.36	67.00	100.00
Sun et al. (2022)	Clinical	DSM‐IV	60	50	China	Lab‐based	Non‐social	28.03	70.00	100.00
Thompson et al. (2017)	Subclinical	BDI	56	56	China	Lab‐based	Non‐social	20.28	70.54	100.00
	Clinical	DSM‐IV	32	21	USA	ESM		31.08	62.17	11.21
Wu et al. (2017)	Clinical	DSM‐IV	41	39	USA	ESM		33.65	80.00	39.21
Yuan and Kring (2009)	Subclinical	BDI	36	36	USA	Lab‐based	Non‐social	19.54	61.10	75.00
Zetsche et al. (2019)	Clinical	DSM‐IV	30	37	Germany	Lab‐based		35.00	82.09	
Zhang et al. (2021)	Subclinical	PHQ‐9	30	34	China	Lab‐based		23.01	71.86	100.00

Abbreviations: % Non‐Caucasian, percentage of non‐Caucasian participants; %Female, percentage of females; BDI, Beck Depression Inventory; CSAS, Chapman Social Anhedonia Scale; DSM, Diagnostic and Statistical Manual of Mental Disorders; ESM, experience sampling method; Lab‐based, laboratory‐based task; PHQ‐9, Patient Health Questionnaire‐9; RSAS, Revised Social Anhedonia Scales; SPQ, Schizotypal Personality Questionnaire.

For anticipated pleasure in the schizophrenia spectrum, we analyzed the data gathered from 20 independent studies, comprising 37 effect sizes, 761 people with schizophrenia and subclinical individuals, and 703 controls. For anticipated displeasure, we included 10 independent studies, comprising 20 effect sizes, 357 people with schizophrenia and subclinical individuals, and 412 controls.

For anticipated pleasure in participants with depression, two studies recruited the same sample (Marroquín et al. 2013, Marroquín 2015), and thus were regarded as one independent study only. Therefore, we analyzed the data gathered from 11 independent studies, comprising 21 effect sizes, 601 people with depression and subclinical individuals, and 561 controls. For anticipated displeasure in depression, we included 8 independent studies, comprising 15 effect sizes, 500 people with depression and subclinical individuals, and 454 controls.

### 3.2 Overall Effect Sizes

#### 3.2.1 Schizophrenia Spectrum Versus Controls

**FIGURE 2:**
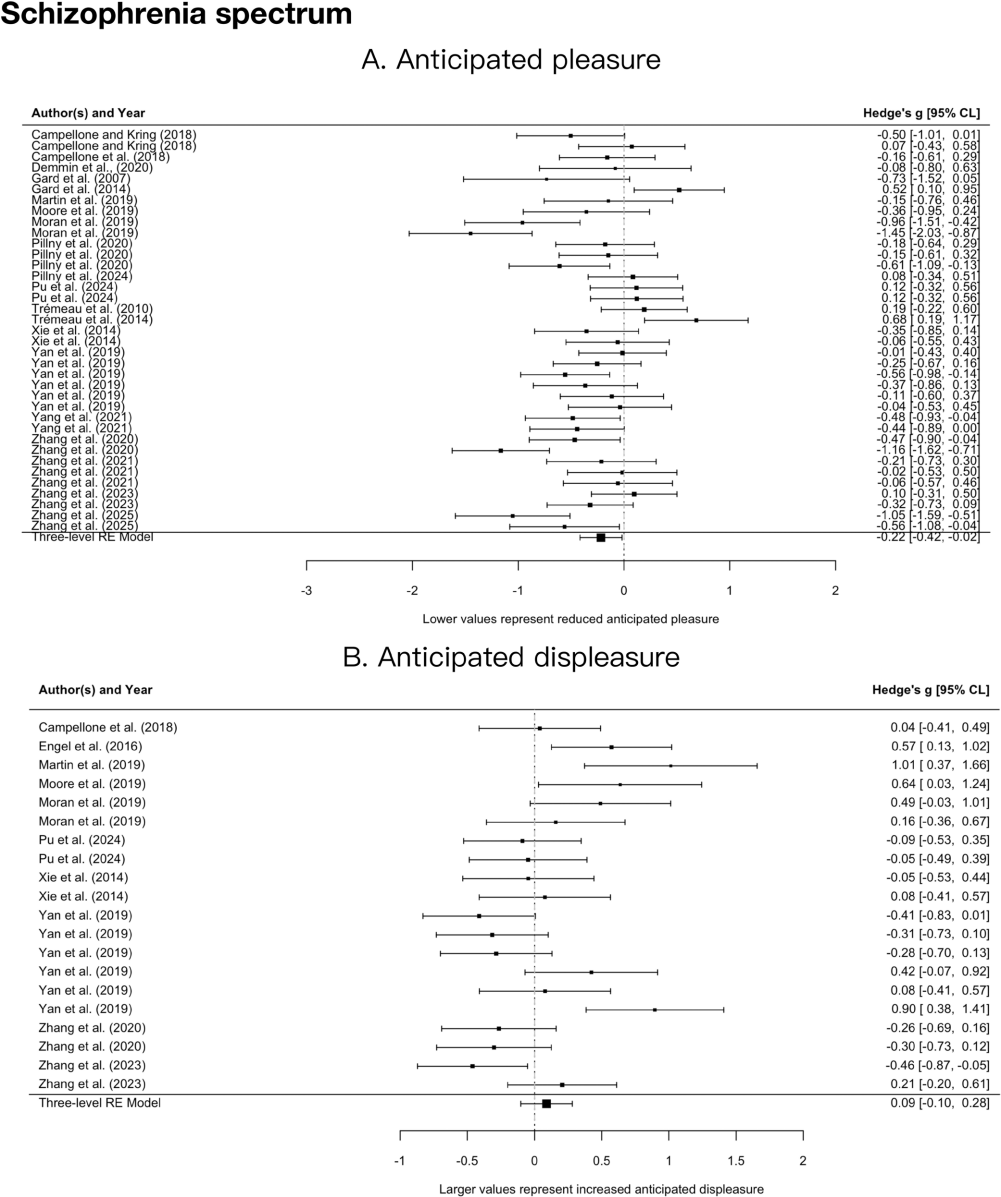
Forest plot between clinical/subclinical samples with schizophrenia and healthy controls. (A) Anticipated pleasure. (B) Anticipated displeasure. Multiple representations of the same study occur where more than one effect size was extracted, with each occurrence representing the extraction of one effect size.

As shown in Figure 2, people with schizophrenia and subclinical individuals reported lower anticipated pleasure than controls, with a small sized effect (*g* = −0.22, 95% CI = [−0.42, −0.02], *p* = 0.032) and substantial heterogeneity (*Q*_*E*_(36) = 106.16, *p* < 0.001). However, they reported similar levels of anticipated displeasure compared with controls, with a nonsignificant effect (*g* = 0.09, 95% CI = [−0.10, 0.28], *p* = 0.345) and substantial heterogeneity (*Q*_*E*_(19) = 55.27, *p* < 0.001).

#### 3.2.2 Depression Versus Controls

**FIGURE 3:**
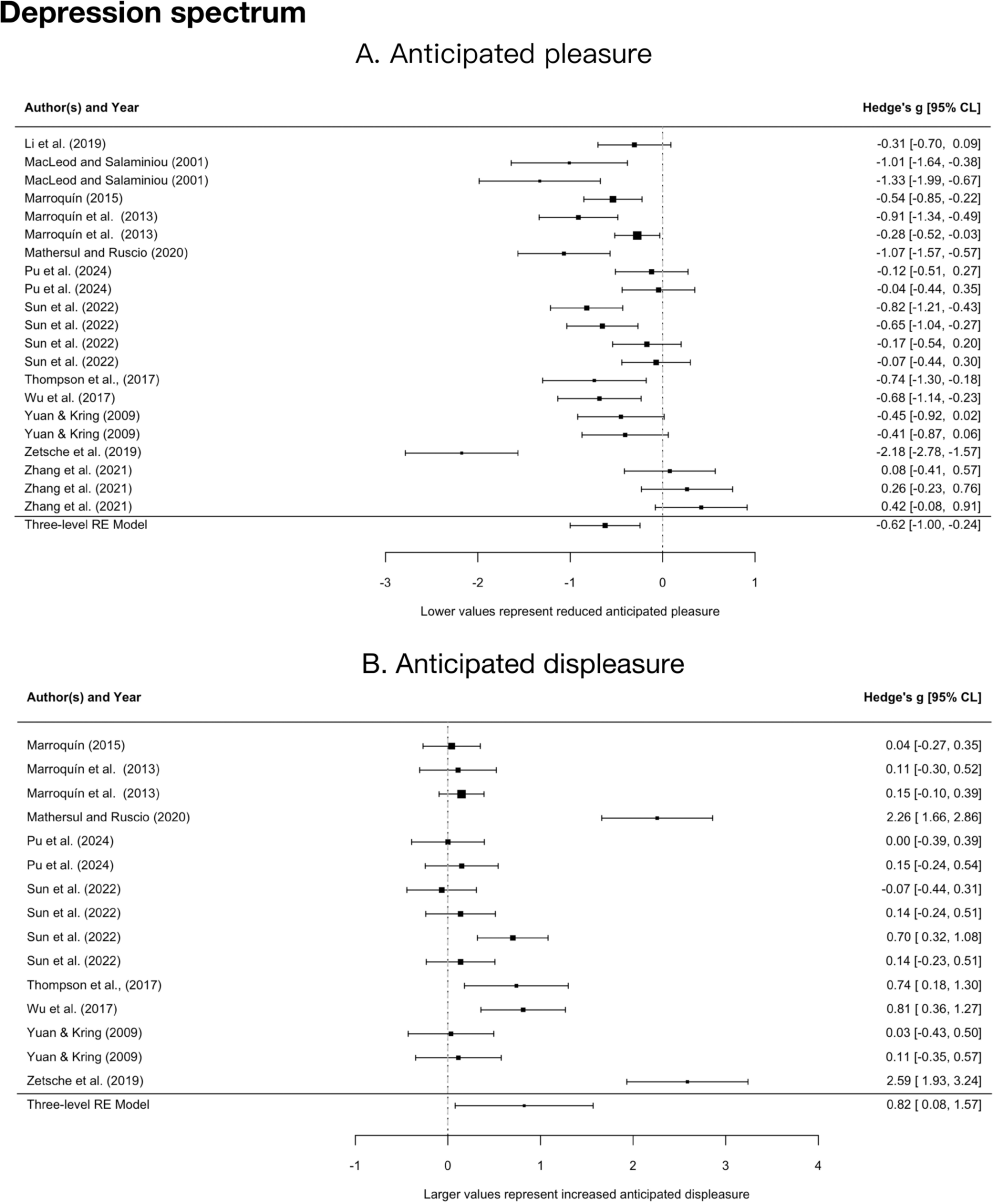
Forest plot between clinical/subclinical samples with depression and healthy controls. (A) Anticipated pleasure. (B) Anticipated displeasure. Multiple representations of the same study occur where more than one effect size was extracted, with each occurrence representing the extraction of one effect size.

As shown in Figure 3, people with depression and subclinical individuals reported lower anticipated pleasure than controls, with a moderate‐to‐large sized effect (*g* = −0.62, 95% CI = [−1.00, −0.25], *p* = 0.003) and substantial heterogeneity (*Q*_*E*_(20) = 97.71, *p* < 0.001). They also reported higher anticipated displeasure than controls, with a large sized effect (*g* = 0.82, 95% CI = [0.08, 1.57], *p* = 0.033) and substantial heterogeneity (*Q*_*E*_(14) = 114.37, *p* < 0.001).

#### 3.2.3 Schizophrenia Versus Depression

Applying the three‐level analyses, we found no significant difference in the effect sizes between schizophrenia and depression, neither for the anticipated pleasure (*F*(1, 56) = 1.73, *p* = 0.194), nor for anticipated displeasure (*F*(1, 33) = 2.54, *p* = 0.120).

### 3.3 Studies on Schizophrenia

#### 3.3.1 Variance of the Overall Effect Size

For anticipated pleasure, the variance at the within‐study level was not significant (estimate = 0.01, *p* = 0.278), but the variance at the between‐study level was significant (estimate = 0.14, *p* = 0.004). Follow‐up analyses found that the degrees of variance at the sampling level, the within‐study level, and the between‐study level were 28.40%, 5.79%, and 65.80%, respectively. Thus, 28.40% of the variance was explained by the samples, indicating that further exploration of potential moderators was needed.

For anticipated displeasure, the variance at the within‐study level was significant (estimate = 0.11, *p* = 0.001), but the variance at the between‐study level was not significant (estimate < 0.01, *p* = 0.050). Follow‐up analyses found that the degrees of variance at the sampling level, the within‐study level, and the between‐study level were 33.45%, 66.55%, and 5.11%, respectively. Thus, 33.45% of the variance was explained by the samples, indicating further exploration of potential moderators was needed.

#### 3.3.2 Moderator Analyses

For anticipated pleasure, sociality of anticipated stimuli was a significant moderator (see Table S1). As shown in Table S2, compared with non‐social stimuli, social stimuli predicted larger deficits of anticipated pleasure (*k* = 29, *β* = 0.27, 95% CI = [0.01, 0.53], SE = 0.13, *p* = 0.041).

For anticipated displeasure, two moderators (study region and % Non‐Caucasian) were significant (see Tables S2 and S3). Specifically, compared with Asian studies, North American studies had larger deficits in anticipated displeasure (*k* = 19, *β* = 0.48, 95% CI = [0.07, 0.89], SE = 0.20, *p* = 0.024). A higher percentage of non‐Caucasian participants predicted smaller deficits in anticipated displeasure (*k* = 19, *β* = −0.01, 95% CI = [−0.02, −0.01], SE = 0.01, *p* = 0.048). Then, we constructed a multiple‐moderator model that included the significant moderators. An omnibus test showed no significant results (*F*(2, 16) = 3.07, *p* = 0.074). Separate moderator analyses of clinical and subclinical samples can be found in Appendix S1: Section 3.

#### 3.3.3 Publication Bias

**FIGURE 4:**
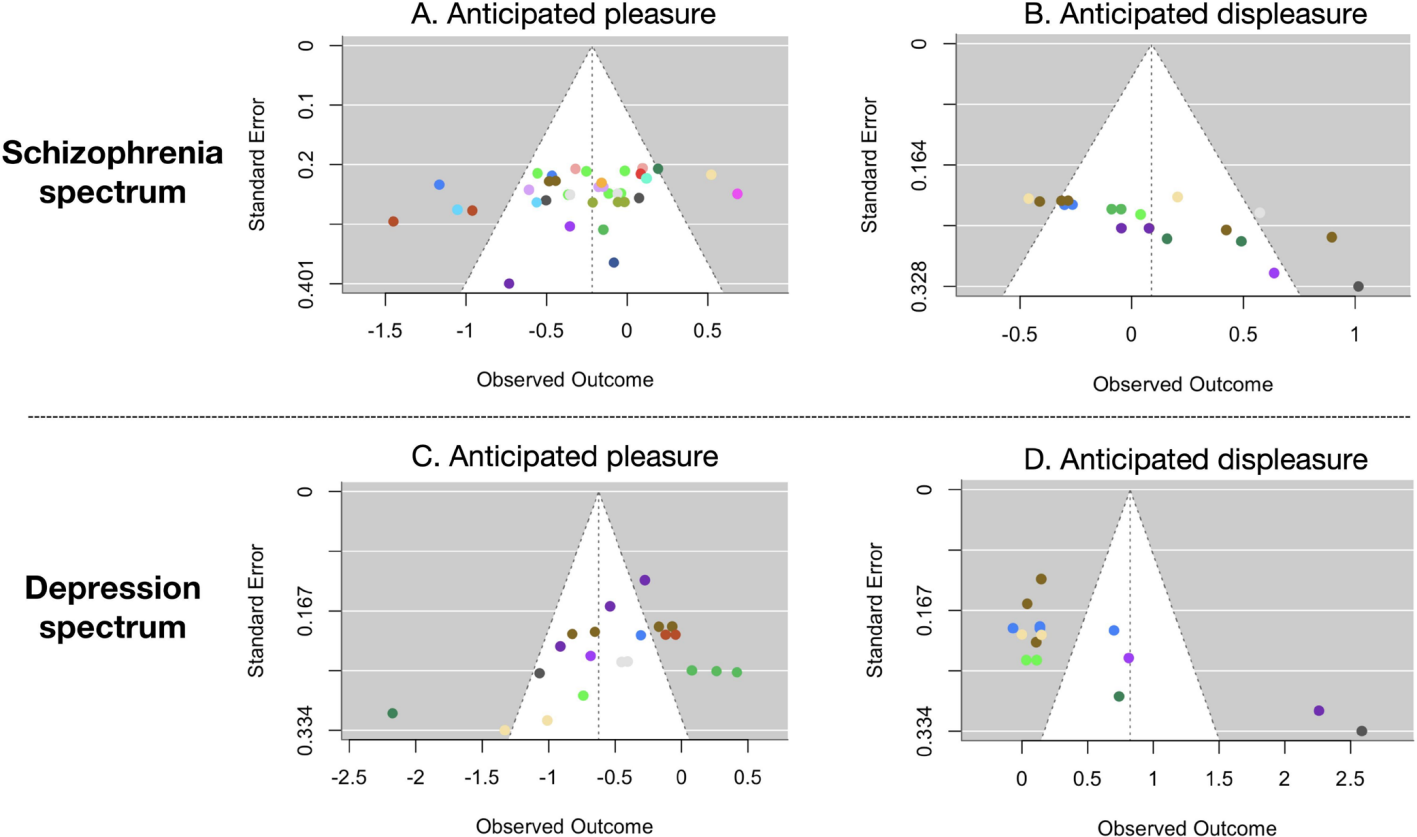
Funnel plot. (A) Anticipated pleasure between clinical/subclinical samples with schizophrenia and healthy controls. (B) Anticipated displeasure between clinical/subclinical samples with schizophrenia and healthy controls. (C) Anticipated pleasure between clinical/subclinical samples with depression and healthy controls. (D) Anticipated displeasure between clinical/subclinical samples with depression and healthy controls. Points of the same color represent the same study.

The funnel plot for anticipated pleasure is illustrated in Figure 4A. The Egger's test did not find any significant asymmetry (*z* = −1.14, *p* = 0.255), suggesting the absence of publication bias.

The funnel plot for anticipated displeasure is illustrated in Figure 4B. The Egger's test showed significant asymmetry (*z* = 5.00, *p* < 0.001), suggesting the presence of publication bias. The trim‐and‐fill procedure found an adjusted effect size of *g* = 0.09, 95% CI = [−0.09, 0.27], *p* = 0.332.

### 3.4 Studies on Depression

#### 3.4.1 Variance of the Overall Effect Size

For anticipated pleasure, the variance at the within‐study level (estimate = 0.04, *p* = 0.030) and the between‐study level (estimate = 0.30, *p* = 0.003) were significant. Follow‐up analyses found that the variance at the sampling, the within‐study, and the between‐study levels were 12.05%, 10.54%, and 77.42%, respectively. Thus, 12.05% of the variance was explained by the samples, indicating that further exploration of potential moderators was needed.

For anticipated displeasure, the variance at the within‐study level was not significant (estimate = 0.01, *p* = 0.312), but the variance at the between‐study level was significant (estimate = 0.91, *p* < 0.001). Follow‐up analyses found that variance at the sampling, within‐study, and between‐study levels was 4.27%, 1.14%, and 94.59%, respectively. Thus, 4.27% of the variance was explained by the samples, indicating further exploration of potential moderators was needed.

#### 3.4.2 Moderator Analyses

For anticipated pleasure, five moderators (clinical status, study region, age, % Non‐Caucasian, and depressive symptom) were significant (see Table S4 and Table S5). Specifically, compared with subclinical individuals, people with clinical depression showed larger deficits in anticipated pleasure (*k* = 21, *β* = −0.74, 95% CI = [−1.08, −0.41], SE = 0.16, *p* < 0.001). Compared with Asian studies, North American studies had larger deficits in anticipated pleasure (*k* = 18, *β* = −0.49, 95% CI = [−0.90, −0.08], SE = 0.20, *p* = 0.024). Older age (*k* = 19, *β* = −0.05, 95% CI = [−0.07, −0.02], SE = 0.01, *p* = 0.002) and more severe depressive symptoms (*k* = 15, *β* = −0.05, 95% CI = [−0.07, −0.03], SE = 0.01, *p* < 0.001) predicted larger deficits in anticipated pleasure, while a higher percentage of non‐Caucasian participants predicted smaller deficits in anticipated pleasure (*k* = 16, *β* = 0.01, 95% CI = [0.01, 0.02], SE = 0.01, *p* = 0.017). Then, we constructed a multiple‐moderator model that included the significant moderators. An omnibus test showed no significant results (*F*(5, 4) = 3.09, *p* = 0.148).

For anticipated displeasure, two moderators (age and depressive symptoms) were significant (see Table S6). Older age (*k* = 13, *β* = 0.10, 95% CI = [0.04, 0.16], SE = 0.03, *p* = 0.006) and more severe depressive symptoms (*k* = 12, *β* = 0.08, 95% CI = [0.03, 0.14], SE = 0.02, *p* = 0.005) predicted larger deficits in anticipated displeasure. Then, we constructed a multiple‐moderator model that included the significant moderators. An omnibus test showed no significant results (*F*(2, 7) = 4.53, *p* = 0.055). Additional analysis of moderators is shown in Appendix S1: Section 3.

#### 3.4.3 Publication Bias

The funnel plot for anticipated pleasure is illustrated in Figure 4C. The Egger's test found significant asymmetry (*z* = −2.33, *p* = 0.020), suggesting the presence of publication bias. The trim‐and‐fill procedure found an adjusted effect size of *g* = −0.50, 95% CI = [−0.74, −0.27], *p* < 0.001.

The funnel plot for anticipated displeasure is illustrated in Figure 4D. The Egger's test showed significant asymmetry (*z* = 4.58, *p* < 0.001), suggesting the presence of publication bias. The trim‐and‐fill procedure found an adjusted effect size of *g* = 0.50, 95% CI = [0.11, 0.89], *p* = 0.012.

## 4 Discussion

This meta‐analysis study summarized the current evidence for anticipated pleasure and displeasure in clinical and subclinical samples of schizophrenia and depression. Our findings suggested that clinical and subclinical samples of the schizophrenia spectrum reported less anticipated pleasure than controls, with a small‐sized effect. However, their anticipated displeasure was similar to controls. Clinical and subclinical samples of depression reported less anticipated pleasure and higher anticipated displeasure than controls, with moderate‐to‐large effect sizes. Moderator analyses showed that, in the schizophrenia spectrum, heterogeneity of anticipated pleasure was explained by sociality of anticipated stimuli. For the participants with depression, higher severity of depressive symptoms were associated with larger between‐group effects of anticipated pleasure and displeasure. After taking publication bias into consideration, the significant between‐group effects remained of a similar magnitude.

Our findings of reduced anticipated pleasure in the schizophrenia spectrum were consistent with previous meta‐analytic findings on anticipatory pleasure (Hallford and Sharma 2019; Visser et al. 2020). The influence of sociality provided valuable insights to the nature of the anticipated pleasure deficit in the schizophrenia spectrum. Compared with non‐social stimuli, social stimuli predicted larger deficits in anticipated pleasure. Previous studies found that both clinical and subclinical populations of the schizophrenia spectrum exhibited social‐related impairment in anticipated pleasure or reward processing (Catalano et al. 2018; Chan et al. 2016; Hooker et al. 2014; Lee et al. 2018; Yin et al. 2015; Zhang et al. 2025, 2020). While the impairment of anticipated social pleasure was moderate, it still has potential as a screening and prevention target for people with schizophrenia spectrum disorders. Moreover, behavioral activation approaches (Jacobson et al. 2001), tailored to early‐stage cultivation of engagement in and anticipation of socially rewarding activities, may help foster and maintain intact anticipated social pleasure. This proactive approach could lead to more effective and efficient prevention outcomes by mitigating the onset or progression of anticipated pleasure deficits associated with schizophrenia spectrum disorders.

Negative symptom severity did not emerge as a strong moderator in the schizophrenia spectrum. This finding may be related to several reasons. First, the non‐significant findings might be attributable to insufficient reporting of clinical symptoms in previous studies, which undermined the power of the analysis. Second, our findings may indicate that negative symptoms do not influence the variance of anticipated emotions, which is consistent with a previous study (Visser et al. 2020). Finally, it may also reflect a lack of coherence between clinical interviews (observer‐rated assessments) and self‐reports (questionnaires, laboratory‐based tasks, or experience sampling tasks), as noted in previous studies (Durand et al. 2015).

Participants with depression reported less anticipated pleasure and higher anticipated displeasure than controls, both with moderate‐to‐large sized effects. Such deficits in affective forecasting may lead to a diminished sense of positive anticipation and motivation in participants with depression, thereby adversely impacting their daily functioning and overall quality of life. Depressed patients exhibit aberrant neural mechanisms in anticipating both positive (Geugies et al. 2022; Keren et al. 2018) and negative (Furman and Gotlib 2016; Rosenblau et al. 2012) stimuli, which might underlie their diminished anticipated pleasure and elevated anticipated displeasure. Compared with subclinical individuals, clinical individuals with depression showed larger deficits in anticipated pleasure. Consistent with a previous study, more severe depressive symptoms were a significant moderator (Gandhi et al. 2022), predicting larger deficits in anticipated pleasure and displeasure. These findings suggested that deficits in anticipated emotions may serve as a marker for the presence of a depressive episode. Identifying individuals with early signs of affective forecasting deficits could allow for early prevention of the progressive development of a depressive episode.

As for the moderator analyses, older age predicted larger deficits in participants with depression, both for anticipated pleasure and anticipated displeasure. In elderly patients with depression, structural and functional brain changes affect emotional and memory function (Aizenstein et al. 2011; de Asis et al. 2001). This may widen the gap of anticipated emotions with age. Older patients also face more negative life events, which may accumulate and exacerbate depressive symptoms, further impairing affective forecasting. Conversely, healthy elderly individuals generally experience less negative emotion and manage emotions more effectively (Reed and Carstensen 2012). Thus, the growing affective forecasting difference with age might stem from healthier individuals' more positive and less negative expectations. A recent meta‐analysis demonstrated that study type had a moderating effect on findings related to consummatory pleasure, with experience sampling studies showing larger group differences relative to laboratory tasks in the schizophrenia population (Abel et al. 2023). However, the study type did not emerge as a significant moderator in our study. This could potentially be attributed to the limited number of experience sampling studies on anticipated emotions.

Moreover, we found cultural variations of heterogeneity. Specifically, compared with Asian samples, North American samples predicted larger deficits in anticipated displeasure for schizophrenia spectrum, as well as larger deficits in anticipated pleasure for depression. Similarly, a higher percentage of non‐Caucasian participants predicted smaller deficits in anticipated displeasure for schizophrenia spectrum, as well as smaller deficits in anticipated pleasure for depression. North American cultures tend to value individualism and emotional expression, while Asian cultures (mostly consisting of non‐Caucasian individuals) often emphasize collectivism and emotional restraint. This may lead North Americans to report more extreme emotional experiences, both positive and negative, while Asians may report more moderate emotions (Tsai et al. 2006), which might influence the cultural variations in anticipated emotions. A meta‐analysis indicated that treatments tailored specifically for Asian populations (e.g., Chinese Americans) showed the largest post‐treatment effects of mental health interventions, and those with no cultural tailoring or non‐Asian tailoring showing the smallest effects (Huey and Tilley 2018). Future research should consider these cultural factors to better understand and address deficits of affective forecasting in different populations.

During study selection, we found many correlational studies investigating the relationship between the severity of psychopathology and the intensity of anticipated pleasure or displeasure. Although these studies did not meet our inclusion criteria, they still provided valuable insights. For example, a recent study involving subclinical traits of schizophrenia showed that the interpersonal features of schizotypal traits were associated with reduced anticipated pleasure for positive social events, but not for non‐social events (Zhang et al. 2023). Furthermore, most studies have found that more severe depressive symptoms were associated with lower anticipated pleasure (Anderson et al. 2023; Hughes et al. 2022; Wenze and Gunthert 2018; Wenze et al. 2013; Zetsche et al. 2019), and with stronger anticipated displeasure (Wenze et al. 2013, 2012; Zetsche et al. 2019). The findings of these correlational studies provide further support for our findings in the current meta‐analysis.

When comparing effect sizes between samples in schizophrenia and depression, no significant difference could be found. In our study, subclinical individuals with schizophrenia primarily exhibited negative symptoms, rather than positive symptoms. Thus, the relevant findings need to be further validated in populations with subthreshold positive symptoms. Furthermore, while the schizophrenia and depression populations show statistically similar overall effect sizes, there are differences in the size and pattern of the effect size compared between the two groups and the healthy control group. This suggests a potential distinction in the qualitative nature of their impairments, despite a quantitatively comparable deviation from the norm. Elucidating these divergent mechanisms requires an understanding of the underlying neurotransmitter dynamics and aberrant neural circuitry, as highlighted in a prior study (Lambert et al. 2018).

Moreover, activating and maintaining cognitive representations of experience are considered crucial for anticipating positive affect and reward (Kring and Caponigro 2010). Several meta‐analyses showed that both people with schizophrenia and depression encountered difficulties in mentally simulating possible future events (Gamble et al. 2019; Hallford et al. 2018), as well as retrieving memory content needed to construct these events (Berna et al. 2015; Sumner et al. 2010). It may be interesting to examine whether difficulty in generating specific mental simulations of future events might co‐vary with anticipated emotions in predicting psychiatric symptoms over time.

This study has several limitations. First, most moderator analyses were conducted in subsets of samples or not performed due to the limited number of studies, thereby reducing the statistical power and generalizability of our findings. Second, our focus was solely on the anticipated dimension. Future studies could explore different temporal distances of emotions, such as consummatory and remembered emotions, to provide a more comprehensive review. Third, the publication bias might limit the generalizability of findings, but our adjusted effect size yielded similar results. Fourth, our meta‐analysis only included two psychiatric disorders. Future studies could encompass a more comprehensive range of psychiatric disorders. Finally, given the current predominance of self‐report methodologies in investigating anticipated anhedonia among clinical and subclinical populations, the present meta‐analysis drew primarily on self‐report studies. Future studies should employ neuroimaging‐based findings to examine neural activation patterns associated with anticipated hedonic processing deficits.

## 5 Conclusion

We provided evidence for deficits in anticipated pleasure in clinical and subclinical samples of schizophrenia and depression. For schizophrenia, heterogeneity was partially explained by the sociality of anticipated stimuli. For depression, it was explained by the severity of depressive symptoms. Clinical and subclinical samples of schizophrenia showed no deficit of anticipated displeasure, while clinical and subclinical samples of depression exhibited higher anticipated displeasure than controls. The heterogeneity of depression studies was explained by depressive symptoms. These findings provided comprehensive estimates of both anticipated pleasure and displeasure in schizophrenia and depression, highlighted the moderating effects, and suggested possible prevention or screening targets.

## Funding

This work was supported by Key Program of the Natural Science Foundation of China, 32430042; Philip K.H. Wong Foundation.

## Conflicts of Interest

The authors declare no conflicts of interest.

## Supporting information


**Appendix S1:** pchj70094‐sup‐0001‐Supinfo.docx.
**Section 1:** Calculation methods for symptom severity.
**Section 2:** Detailed information for statistical analysis.
**Section 3:** Additional analysis.
**Table S1:** Anticipated pleasure for people with schizophrenia and subclinical counterparts: The *Q*_*E*_ statistics testing residual heterogeneity and the Omnibus Test reflecting the effects of the moderators.
**Table S2:** Significant moderators for people with schizophrenia and subclinical counterparts.
**Table S3:** Anticipated displeasure for people with schizophrenia and subclinical counterparts: The *Q*_*E*_ statistics testing residual heterogeneity and the Omnibus Test reflecting the effects of the moderators.
**Table S4:** Anticipated pleasure for people with depression and subclinical counterparts: The *Q*_*E*_ statistics testing residual heterogeneity and the Omnibus Test reflecting the effects of the moderators.
**Table S5:** Significant moderators for people with depression and subclinical counterparts (anticipated pleasure).
**Table S6:** Anticipated displeasure for people with depression and subclinical counterparts: the *Q*_*E*_ statistics testing residual heterogeneity and the Omnibus Test reflecting the effects of the moderators.

## Data Availability

The data that support the findings of this study are openly available in Science Data Bank at https://doi.org/10.57760/sciencedb.11320.
