# Supplementary material for: Transdiagnostic Comparison of Anticipated Hedonic Deficits: A Three‐Level Meta‐Analysis Across Schizophrenia, Depression, and Their Subclinical Individuals
Source: Psych J. 2026 Apr 2;15(2):e70094. doi: 10.1002/pchj.70094 (PMC13045473; doi:10.1002/pchj.70094)
Supplement: Supplementary file 1 — Appendix S1: pchj70094‐sup‐0001‐Supinfo.docx. Section 1: Calculation methods for symptom severity. Section 2: Detailed information for statistical analysis. Section 3: Additional analysis. Table S1: Anticipated pleasure for people with schizophrenia and subclinical counterparts: The Q E statistics testing residual heterogeneity and the Omnibus Test reflecting the effects of the moderators. Table S2: Significant moderators for people with schizophrenia and subclinical counterparts. Table S3: Anticipated displeasure for people with schizophrenia and subclinical counterparts: The Q E statistics testing residual heterogeneity and the Omnibus Test reflecting the effects of the moderators. Table S4: Anticipated pleasure for people with depression and subclinical counterparts: The Q E statistics testing residual heterogeneity and the Omnibus Test reflecting the effects of the moderators. Table S5: Significant moderators for people with depression and subclinical counterparts (anticipated pleasure). Table S6: Anticipated displeasure for people with depression and subclinical counterparts: the Q E statistics testing residual heterogeneity and the Omnibus Test reflecting the effects of the moderators. [file PCHJ-15-e70094-s001.docx]

**Appendix S1**

**Section 1: Calculation methods for symptom severity**

For negative symptom severity, the total scores from the Scale for the Assessment of Negative Symptoms (SANS) and the Brief Negative Symptom Scale (BNSS) were converted to their equivalents on the Positive and Negative Syndrome Scale (PANSS) negative subscale using the validated conversion equations (Kott & Daniel, 2018; van Erp et al., 2014). To our knowledge, no validated method is available for converting the Clinical Assessment Interview for Negative Symptoms (CAINS) scores to the PANSS scores. Therefore, the CAINS scores could not be included in the meta-regression analyses.

Moreover, due to the absence of commonly used conversion methods for different scales of depressive symptoms (Hawley et al., 2013), we followed a previous method (Gandhi et al., 2022) to transform scores from different depressive symptom scales into proportions of their respective maximum scores. Specifically, we divided the mean values of depressive symptoms by the maximum score.

**Section 2: Detailed information for statistical analysis**

Extracting multiple effect sizes from the same study might violate the assumption of independence required for traditional meta-analytic approaches (Assink & Wibbelink, 2016). To address the interdependency of effect sizes, we applied a three-level random-effects model to synthesize effect sizes across studies and conduct moderator analyses. By adopting the three-level meta-analytic model, we could preserve all information and achieve maximum statistical power (Van den Noortgate et al., 2013).

Specifically, in the three-level meta-analytic models (Cheung, 2014), three different sources of variance were modeled: (1) sampling variance of effect sizes (Level 1), (2) variance between effect sizes extracted from the same study (Level 2), and (3) variance between studies (Level 3). The three-level analysis was conducted in the following steps. First, the overall mean effect sizes were estimated to assess the deficit of anticipated pleasure and displeasure in clinical and subclinical groups, relative to controls. Second, a likelihood-ratio test was applied to assess the between-study (Level 3) and the within-study (Level 2) heterogeneity. When we found evidence for heterogeneity in effect sizes, moderation analyses were performed, based on the 75% rule (Hunter & Schmidt, 1990). It has been suggested that, when less than 75% of the total variance can be attributed to random sampling error (Level 1), heterogeneity at Level 2 (within studies) and Level 3 (between studies) can be considered substantial. In that case, we further performed moderator analyses. To enhance reliability, we only conducted moderator analyses when the category contained at least three studies (Morgan et al., 2018). Third, a multiple-moderator model including all the significant moderators was performed to control for the associations among moderators.

**Section 3: Additional analysis**

We repeated the moderator analysis separately for the clinical and subclinical samples to explore potential significant moderators. The results showed that, between schizophrenia clinical group and control group, older age was associated with larger between-group effect of anticipated displeasure (k = 10, β = 0.05, 95% *CI* = [0.01, 0.11], SE = 0.02, *p* = 0.047), and higher proportion of non-Caucasian participants was associated with smaller between-group effect of anticipated displeasure (k = 9, β = -0.02, 95% *CI* = [-0.03, -0.01], SE = 0.01, *p* = 0.028). Then, we constructed a multiple-moderator model that included the significant moderators. An omnibus test showed significant results (*F*(2,6) = 8.91, *p* = 0.016), suggesting that at least one of the regression coefficients of the moderators significantly deviated from zero. Such results suggested that age (k = 9, β = 0.04, 95% *CI* = [0.01, 0.08], SE = 0.02, *p* = 0.043) had unique moderating effects. Between schizophrenia subclinical group and control group, older age was associated with smaller between-group effect of anticipated displeasure (k = 10, β = -0.73, 95% *CI* = [-1.45, -0.01], SE = 0.31, *p* = 0.048). For the comparison between depression subclinical group and control group, lower proportion of non-Caucasian participants (k = 11, β = -0.01, 95% *CI* = [-0.02, -0.01], SE = 0.01, *p* = 0.039) and more severe depressive symptoms (k = 10, β = -0.05, 95% *CI* = [-0.09, -0.01], SE = 0.02, *p* = 0.018) predicted larger between-group effect in anticipated pleasure. Then, we constructed a multiple-moderator model that included the significant moderators. An omnibus test showed no significant results (F(2,5) = 3.14, p = 0.131).

We also repeated the moderator analysis of sample characteristics (i.e., age, % of female, % of non-Caucasian, and education level), separately for the clinical/subclinical group and control group. Results showed that higher percentage of non-Caucasian in the control group was associated with smaller effect of anticipated displeasure between schizophrenia group and control group (k = 19, β = -0.02, 95% *CI* = [-0.03, -0.01], SE = 0.01, *p* = 0.012). Older age in the clinical/subclinical depression group was associated with larger effect of anticipated displeasure between depression group and control group (k = 10, β = 0.10, 95% *CI* = [0.02, 0.17], SE = 0.03, *p* = 0.016).

**Table S1. Anticipated pleasure for people with schizophrenia and subclinical counterparts: the *Q_E_* statistics testing residual heterogeneity and the Omnibus Test reflecting the effects of the moderators**

| **Moderator** | ***Q_E_ (df)*** | ***p*** | **Omnibus test** | ***p*** |
| --- | --- | --- | --- | --- |
| **Study characteristics** |  |  |  |  |
| Clinical status | 102.45 (35) | <0.001 | *F*(1,35) = 0.32 | 0.578 |
| Region | 98.76 (31) | <0.001 | *F*(1,31) = 0.68 | 0.417 |
| NOS score | 95.40 (35) | <0.001 | *F*(1,35) = 3.26 | 0.080 |
| Type of task | 103.38 (33) | <0.001 | *F*(1,33) = 0.01 | 0.997 |
| Sociality | 83.57 (27) | <0.001 | *F*(1,27) = 4.62 | **0.041** |
| Hypotheticality | 103.12 (35) | <0.001 | *F*(1,35) = 0.64 | 0.429 |
| Temporal distance | 89.50 (33) | <0.001 | *F*(1,33) = 1.99 | 0.168 |
| **Sample characteristics** |  |  |  |  |
| Age | 100.70 (35) | <0.001 | *F*(1,35) = 1.11 | 0.299 |
| %Female | 94.19 (35) | <0.001 | *F*(1,35) = 3.13 | 0.086 |
| %Non-Caucasian | 83.62 (28) | <0.001 | *F*(1,28) = 0.38 | 0.542 |
| Education year | 93.32 (25) | <0.001 | *F*(1,25) = 0.78 | 0.384 |
| **Clinical characteristics** |  |  |  |  |
| Duration of illness | 20.96 (8) | 0.007 | *F*(1,8) = 0.12 | 0.734 |
| %Typical | 46.27 (8) | <0.001 | *F*(1,8) = 0.73 | 0.417 |
| Doses | 37.50 (12) | <0.001 | *F*(1,12) = 4.57 | 0.054 |
| Negative symptom | 21.97 (10) | 0.015 | *F*(1,10) = 3.56 | 0.089 |

*Note.* Statistically significant results are in bold. Clinical status: clinical or subclinical samples; Type of task: questionnaires, laboratory-based task, or experience sampling task; Sociality (of anticipated stimuli): social or non-social future events; Hypotheticality (of anticipated stimuli): hypothetical or actual future events; Temporal distance (of anticipated stimuli): immediate (next few hours) or distant (next day or month); %Female: percentage of females; %Non-Caucasian: percentage of non-Caucasian participants; %Typical: proportion of people with schizophrenia prescribed typical antipsychotics; Doses: chlorpromazine equivalent doses.

**Table S2. Significant moderators for people with schizophrenia and subclinical counterparts**

| **Moderator** | *k* | *ES* | *SE* | *t* | *95% CI (ES)* | *p* |
| --- | --- | --- | --- | --- | --- | --- |
| **Anticipated pleasure** |  |  |  |  |  |  |
| Sociality | 29 |  |  |  |  |  |
| Social AF | 11 | -0.40 | 0.14 | -2.95 | [-0.68, -0.12] | **0.007** |
| Non-social AF | 18 | -0.13 | 0.13 | 1.03 | [-0.40, 0.13] | 0.133 |
| ΔSlope of social (vs non-social) |  | 0.27 | 0.13 | 2.15 | [0.01, 0.53] | **0.041** |
|  |  |  |  |  |  |  |
| **Anticipated displeasure** |  |  |  |  |  |  |
| Region | 19 |  |  |  |  |  |
| Asian | 14 | -0.06 | 0.09 | -0.61 | [-0.25, 0.14] | 0.549 |
| North American | 5 | 0.43 | 0.17 | 2.49 | [0.07, 0.79] | **0.023** |
| ΔSlope of Asian (vs. North American) |  | 0.48 | 0.20 | 2.48 | [0.07, 0.89] | **0.024** |

Note. Statistically significant results are in bold. *ES*=effect size. *CI* = confidence interval. Sociality (of anticipated stimuli): social or non-social future events; Clinical status: clinical or subclinical samples.

**Table S3. Anticipated displeasure for people with schizophrenia and subclinical counterparts: the *Q_E_* statistics testing residual heterogeneity and the Omnibus Test reflecting the effects of the moderators**

| **Moderator** | ***Q_E_ (df)*** | ***p*** | **Omnibus test** | ***p*** |
| --- | --- | --- | --- | --- |
| **Study characteristics** |  |  |  |  |
| Clinical status | 54.87 (18) | <0.001 | *F*(1,18) = 0.07 | 0.795 |
| Region | 37.88 (17) | 0.003 | *F*(1,17) = 6.15 | **0.024** |
| NOS score | 54.97 (18) | <0.001 | *F*(1,18) = 0.22 | 0.646 |
| Sociality | 49.55 (17) | <0.001 | *F*(1,17) = 0.02 | 0.889 |
| **Sample characteristics** |  |  |  |  |
| Age | 52.58 (18) | <0.001 | *F*(1,18) = 1.44 | 0.246 |
| %Female | 54.13 (18) | <0.001 | *F*(1,18) = 0.20 | 0.661 |
| %Non-Caucasian | 40.53 (17) | 0.001 | *F*(1,17) = 4.54 | **0.048** |
| Education year | 35.69 (12) | <0.001 | *F*(1,12) = 2.03 | 0.180 |
| **Clinical characteristics** |  |  |  |  |
| Doses | 10.01 (5) | 0.075 | *F*(1,5) = 4.22 | 0.095 |
| Negative symptom | 6.82 (4) | 0.146 | *F*(1,4) = 4.57 | 0.099 |

*Note.* Statistically significant results are in bold. Clinical status: clinical or subclinical samples; Sociality (of anticipated stimuli): social or non-social future events; %Female: percentage of females; %Non-Caucasian: percentage of non-Caucasian participants; Doses: chlorpromazine equivalent doses.

**Table S4. Anticipated pleasure for people with depression and subclinical counterparts: the *Q_E_* statistics testing residual heterogeneity and the Omnibus Test reflecting the effects of the moderators**

*Note.* Statistically significant results are in bold. Clinical status: clinical or subclinical samples; Type of task: questionnaires, laboratory-based task, or experience sampling task; Hypotheticality (of anticipated stimuli): hypothetical or actual future events; Temporal distance (of anticipated stimuli): immediate (next few hours) or distant (next day or month); %Female: percentage of females; %Non-Caucasian: percentage of non-Caucasian participants.

| **Moderator** | ***Q_E_ (df)*** | ***p*** | **Omnibus test** | ***p*** |
| --- | --- | --- | --- | --- |
| **Study characteristics** |  |  |  |  |
| Clinical status | 50.76 (19) | <0.001 | *F*(1,19) = 21.39 | **<0.001** |
| Region | 40.21 (16) | <0.001 | *F*(1,16) = 6.26 | **0.024** |
| NOS score | 86.52 (19) | <0.001 | *F*(1,19) = 0.68 | 0.420 |
| Type of task | 97.09 (19) | <0.001 | *F*(1,19) = 0.03 | 0.864 |
| Hypotheticality | 83.62 (17) | <0.001 | *F*(1,17) = 0.80 | 0.383 |
| Temporal distance | 95.08 (19) | <0.001 | *F*(1,19) = 1.66 | 0.213 |
| **Sample characteristics** |  |  |  |  |
| Age | 53.38 (17) | <0.001 | *F*(1,17) = 13.46 | **0.002** |
| %Female | 90.03 (17) | <0.001 | *F*(1,17) = 0.01 | 0.926 |
| %Non-Caucasian | 30.65 (14) | 0.006 | *F*(1,14) = 7.34 | **0.017** |
| Depressive symptom | 22.44 (11) | 0.021 | *F*(1,11) = 21.69 | **<0.001** |

**Table S5. Significant moderators for people with depression and subclinical counterparts (anticipated pleasure).**

| **Moderator** | *k* | *ES* | *SE* | *t* | *95% CI (ES)* | *p* |
| --- | --- | --- | --- | --- | --- | --- |
| Clinical status | 21 |  |  |  |  |  |
| Clinical individuals | 8 | -1.00 | 0.15 | -6.80 | [-1.31, -0.70] | **<0.001** |
| Subclinical individuals | 13 | -0.26 | 0.14 | -1.95 | [-0.54, 0.02] | **0.020** |
| ΔSlope of clinical (vs. subclinical) |  | -0.74 | 0.16 | -4.62 | [-1.08, -0.41] | **<0.001** |
| Region | 18 |  |  |  |  |  |
| Asian | 10 | -0.14 | 0.14 | -1.06 | [-0.43, 0.15] | 0.307 |
| North American | 8 | -0.63 | 0.14 | -4.50 | [-0.93, -0.33] | **<0.001** |
| ΔSlope of Asian (vs. North American) |  | -0.49 | 0.20 | -2.50 | [-0.90, -0.08] | **0.024** |

Note. Statistically significant results are in bold. *ES*=effect size. *CI*=confidence interval. Clinical status: clinical or subclinical samples.

**Table S6. Anticipated displeasure for people with depression and subclinical counterparts: the *Q_E_* statistics testing residual heterogeneity and the Omnibus Test reflecting the effects of the moderators**

*Note.* Statistically significant results are in bold. Clinical status: clinical or subclinical samples; Hypotheticality (of anticipated stimuli): hypothetical or actual future events; Temporal distance (of anticipated stimuli): immediate (next few hours) or distant (next day or month); %Female: percentage of females; %Non-Caucasian: percentage of non-Caucasian participants.

| **Moderator** | ***Q_E_ (df)*** | ***p*** | **Omnibus test** | ***p*** |
| --- | --- | --- | --- | --- |
| **Study characteristics** |  |  |  |  |
| Clinical status | 92.25 (13) | <0.001 | *F*(1,13) = 2.28 | 0.155 |
| NOS score | 66.56 (13) | <0.001 | *F*(1,13) = 2.26 | 0.157 |
| Hypotheticality | 109.44 (13) | <0.001 | *F*(1,13) = 0.44 | 0.518 |
| Temporal distance | 109.75 (13) | <0.001 | *F*(1,13) = 3.12 | 0.101 |
| **Sample characteristics** |  |  |  |  |
| Age | 68.70 (11) | <0.001 | *F*(1,11) = 11.79 | **0.006** |
| %Female | 87.32 (11) | <0.001 | *F*(1,11) = 3.00 | 0.111 |
| %Non-Caucasian | 54.27 (10) | <0.001 | *F*(1,10) = 1.50 | 0.249 |
| Depressive symptom | 37.20 (10) | <0.001 | *F*(1,10) = 12.59 | **0.005** |

**References**

Assink, M., & Wibbelink, C. J. M. (2016). Fitting three-level meta-analytic models in R: A step-by-step tutorial. *The Quantitative Methods for Psychology*, *12*(3), 154-174.

Cheung, M. W. L. (2014). Modeling dependent effect sizes with three-level meta-analyses: A structural equation modeling approach. *Psychological Methods*, *19*(2), 211-229.

Gandhi, A., Mote, J., & Fulford, D. (2022). A transdiagnostic meta-analysis of physical and social anhedonia in major depressive disorder and schizophrenia spectrum disorders. *Psychiatry Research*, *309*, 114379.

Hawley, C. J., Gale, T. M., Smith, P. S. J., Jain, S., Farag, A., Kondan, R., Avent, C., & Graham, J. (2013). Equations for converting scores between depression scales (MÅDRS, SRS, PHQ-9 and BDI-II): good statistical, but weak idiographic, validity. *Human Psychopharmacology: Clinical and Experimental*, *28*(6), 544-551.

Hunter, J. E., & Schmidt, F. L. (1990). *Methods of meta-analysis: Correcting error and bias in research findings*. Newbury Park: Sage.

Kott, A., & Daniel, D. (2018). An exploratory analysis converting scores between the PANSS and BNSS. *Schizophrenia Bulletin*, *44*(suppl_1), S135-S136.

Morgan, A. J., Ross, A., & Reavley, N. J. (2018). Systematic review and meta-analysis of Mental Health First Aid training: Effects on knowledge, stigma, and helping behaviour. *PloS One*, *13*(5), e0197102.

Van den Noortgate, W., López-López, J. A., Marín-Martínez, F., & Sánchez-Meca, J. (2013). Three-level meta-analysis of dependent effect sizes. *Behavior Research Methods*, *45*(2), 576-594.

van Erp, T. G., Preda, A., Nguyen, D., Faziola, L., Turner, J., Bustillo, J., Belger, A., Lim, K. O., McEwen, S., Voyvodic, J., Mathalon, D. H., Ford, J., Potkin, S. G., & Fbirn. (2014). Converting positive and negative symptom scores between PANSS and SAPS/SANS. *Schizophrenia Research*, *152*(1), 289-294.
